# Convergence and stability analysis of the Extended Infinite Horizon Model Predictive Control

**DOI:** 10.1371/journal.pone.0344964

**Published:** 2026-04-01

**Authors:** Luz A. Alvarez, Diego F. de Bernardini, Christophe Gallesco

**Affiliations:** 1 School of Chemical Engineering, University of Campinas–UNICAMP, Campinas, São Paulo, Brazil; 2 Department of Statistics, Institute of Mathematics, Statistics and Scientific Computation, University of Campinas–UNICAMP, Campinas, São Paulo, Brazil; Aalto University, FINLAND

## Abstract

Model Predictive Control (MPC) is a popular technology to operate industrial systems. It refers to a class of control algorithms that use an explicit model of the system to obtain the control action by minimizing a cost function. At each time step, MPC solves an optimization problem that minimizes the future deviation of the outputs which are calculated from the model. The solution of the optimization problem is a sequence of control inputs, the first of which is actually applied to the system. The optimization process is then repeated at subsequent time steps. In the context of MPC, convergence and stability are fundamental issues. This paper presents a mathematical analysis for convergence and stability of two important controllers: the Extended Infinite Horizon MPC developed by Odloak [Odloak D. Extended robust model predictive control. AIChE J. 2004;50(8): 1824–1836] and the Extended Infinite Horizon MPC with zone control [González AH, Odloak D. A stable MPC with zone control. J Process Control. 2009;19: 110–122]. Our analysis provides tuning strategies that can be implemented in practice, and the mathematical tools we use are intended to serve as a rigorous background for future studies and developments of related MPC approaches.

## Introduction

Model Predictive Control (MPC) originated in the late seventies and has been developed considerably since then [1]. It was originally developed to meet the specialized control needs of petroleum refineries and power plants, but today MPC represents a powerful technology to operate complex dynamic systems, with several industrial applications, including process control, automotive systems, robotics, and energy management. MPC refers to a class of control algorithms that use an explicit model of the system to obtain the control action by minimizing a cost function. The model represents a dynamic relation between system inputs (control actions) and outputs (measurements). The purpose of the model is to predict the future response of the outputs over a prediction horizon. At each time step, an MPC algorithm solves an optimization problem that contains a performance cost function, the predictive model of the system and constraints on inputs and outputs. The solution of this problem is a sequence of inputs, the first input in the optimal sequence is then applied to the system, and the optimization process is repeated at the next time step, incorporating updated output measurements.

Fundamental aspects of any control system are convergence and stability, which together are often called *asymptotic stability*. Convergence refers to the ability of the optimization process to reach a solution that satisfies the control objectives. On the other hand, guarantee of stability is essential to prevent undesirable behaviors such as oscillations, instability, or divergence, which can compromise the performance and safety of the system. Various approaches have been proposed to analyze and ensure the stability of MPC. Since MPC uses a prediction model, the stability of the closed-loop system with MPC is classified in two types: if the prediction model perfectly represents the process system, the stability is nominal, and if there is uncertainty in the prediction model, the stability is robust. An usual method to obtain nominal stability of an MPC closed-loop system is to adopt an infinite prediction horizon. In this sense [2], developed an MPC regulator with infinite prediction horizon and input and output constraints. For stable open-loop systems, the infinite horizon can be reduced to a finite horizon MPC with a terminal weight computed through the solution of a Lyapunov equation. The infinite horizon MPC was later extended to the reference tracking problem by [3]. This work overcame the need to know the system steady state allowing the application to the output-tracking problem and the regulator problem with unknown disturbances, which was one of the major barriers to implement infinite horizon MPC in practice. Furthermore [4], presented an easier and more practical infinite horizon MPC. Following the method of [3], slack variables were added to the optimization problem, allowing a minimal violation of constraints, and keeping the cost function limited for the disturbed system. This feature is important for practical implementation.

In this paper, we consider the Extended Infinite Horizon MPC (EIHMPC) proposed by [4], which is a nominal infinite horizon MPC that considers an Output Prediction-Oriented Model (OPOM). The OPOM is a state-space model arranged in the incremental form of inputs, and it is developed from the analytical form of the step response of the system [3]. The EIHMPC was further developed for zone control, where the outputs are controlled inside zones or ranges instead of fixed set-points [5]. The consideration of the output zones results in additional degrees of freedom left to the MPC optimization problem, which means that all or some inputs are free to be moved to optimal targets that can be defined externally or calculated through a real time optimization (RTO) layer. Over the last 20 years, these controllers have gained attention in the academic community and chemical process industry. For instance, the EIHMPC with zone control served as a basis for subsequent approaches: it was extended to systems with integrating poles [6–8], to integrating systems with optimizing targets [9], to dead time systems [10–14] and to unstable systems [15]. This controller was also formulated in two layers to receive the real time optimization targets, which are the solution of an economic optimization problem [16–18]. Moreover, the EIHMPC has been implemented in the context of process design methodologies that consider simultaneously economic profit, dynamic performance, and process safety [19,20]. On the other hand, control technologies based on the EIHMPC have also been successfully applied in real process industries, especially oil refineries [21–25] as well as pilot scale plants [26,27]. Nowadays, the EIHMPC is part of an in-house advanced control package developed by Petrobras (Petróleo Brasileiro S.A.) and has been implemented in many process units of the main oil refineries of Brazil [28].

Although the EIHMPC and EIHMPC with zone control have been widely implemented, their theoretical foundations concerning asymptotic stability under general conditions have remained incomplete. The original papers only provided intuitive insights on asymptotic stability for the specific case where the gain matrix *D*_0_ is regular [4] and the input horizon *m* is equal to 1 [4,5]. Rigorous and general proofs covering both arbitrary gain matrix and input horizon have been lacking, limiting further theoretical extensions and reliable tuning guidelines. The main objective of this paper is to provide mathematical proofs of asymptotic stability for both controllers with general gain matrix *D*_0_, i.e., not necessarily regular (allowing the input and output vectors to have different dimensions), and general input horizon *m*. Despite the similarities, it is important to strengthen that the EIHMPC approach with fixed set-point is not a particular case of the EIHMPC with zone control. Therefore it is important to present mathematical proofs for each one of them. As the reader may note, the mathematical treatment of the optimization problems of both controllers presents several technical difficulties, such as the construction of suitable weighting matrices *S* and *S*_*u*_ for the corresponding slack variables and the formulation of appropriate control strategies to establish convergence. This reveals the challenge of obtaining rigorous results. As a byproduct of our analysis, for both approaches, we provide explicit expressions for tuning parameters *S* and *S*_*u*_ that can be implemented in practice. Finally, we believe that the proofs presented in this work could be adapted to derived approaches and serve as a mathematical background for future studies and developments.

The paper is organized as follows. In the Extended Infinite Horizon MPC section, we present the OPOM and the EIHMPC formulation, the corresponding convergence and stability theorems and their proofs. In the Extended Infinite Horizon MPC with zone control section, we present the EIHMPC with zone control, the convergence and stability results and their proofs. In the Appendix, we give a recipe to compute the tuning parameter *S* of the EIHMPC, followed by a toy example to illustrate its computation.

### Extended Infinite Horizon MPC

We begin by introducing the Extended Infinite Horizon Model Predictive Control in the Formulation and results section, which is based on the Output Prediction-Oriented Model. The OPOM is a discrete time state-space representation that includes the input increments Δu, the outputs *y*, and the states *x*_*s*_ and *x*_*d*_. Subsequently, we present the cost function *V* of the controller, which contains an infinite-horizon term, along with a terminal state constraint on *x*_*s*_ that includes a slack variable δ.

We assume that the open-loop system described by the OPOM is stable (Assumption 1). Open-loop stability means that the open-loop system converges to any steady state when an input value is applied. It is relevant in practice because if the system is unstable, the EIHMPC cannot be implemented. Under this assumption, and by decomposing the infinite horizon cost *V* into two components, it is possible to reduce the infinite term to a finite horizon term with a terminal weight $\bar{Q}$. Then, the formulation of the corresponding control optimization problem is presented. It includes both the terminal state constraint on *x*_*s*_ and the input constraints. At each time step *k*, the optimization variables are the sequence of input increments $\Delta u_{k}$ and the slack variable $\delta_{k}$.

Next, we introduce Assumption 2, which states that the output reference (also called set-point) *r* is reachable from the input set 𝕌. Here, reachability refers to the capacity of the system to reach *r* from some input within 𝕌. Its relevance relies on the fact that if *r* is unreachable, the output cannot converge to that reference value. In practice, when *r* is unreachable, the input saturates (i.e., reaches the boundary of 𝕌) trying, but not succeeding, to bring the output to the set-point.

Based on Assumptions 1 and 2, Theorems 1 and 2 are established, which address the convergence and stability of the closed-loop system.

### Formulation and results

The EIHMPC considers the following OPOM, which is a discrete time state-space model obtained from the step response [4,5]. For discrete time k≥0, let


$$\begin{array}{c} \left[\begin{array}{c} x_{s}(k+1)\\ x_{d}(k+1) \end{array}]=[\begin{array}{cc} I & 0\\ 0 & F \end{array}][\begin{array}{c} x_{s}(k)\\ x_{d}(k) \end{array}]+[\begin{array}{c} D_{0}\\ D_{d} \end{array}](u(k+1)-u(k)\right) \end{array}$$ (1)

and


$$\begin{array}{c} y(k)=[\begin{array}{cc} I & \Psi \end{array}][\begin{array}{c} x_{s}(k)\\ x_{d}(k) \end{array}], \end{array}$$ (2)

where $x_{s}\in {\mathbb{R}}^{n_{y}}$, $x_{d}\in {\mathbb{C}}^{n_{d}}$, $u\in {\mathbb{R}}^{n_{u}}$, $y\in {\mathbb{R}}^{n_{y}}$, $F\in {\mathbb{C}}^{n_{d}\times n_{d}}$, $D_{0}\in {\mathbb{R}}^{n_{y}\times n_{u}}$, $D_{d}\in {\mathbb{C}}^{n_{d}\times n_{u}}$, $\Psi \in {\mathbb{R}}^{n_{y}\times n_{d}}$ and *I* is the identity matrix of dimension *n*_*y*_. It is worth noting that the matrix *D*_*d*_ considered here corresponds to the matrix *D*^d^*FN* in [4]. In the state equation (1), *x*_*s*_ is called the *static part* of the state of the system, while *x*_*d*_ is called the *dynamic part*. The vector *u* represents the *inputs* of the model and *y* stands for the *outputs*. Matrix *D*_0_ is called the *static gain* of the system. We will not go deeper into the structure of the matrices appearing in the model, because it is not relevant for the present work. The interested reader may consult the above references for more details.

In the infinite horizon MPC setting, we denote by m∈ℕ the input horizon. Since, at each time step k≥1, an optimization problem is solved for *u* over the horizon time interval of size *m*, we introduce the following notation: for j=0,1,…,m−1, we define Δu(j|k) as the *j*-th move of the input solution of the optimization problem (to be defined below) at time step *k*. For the other variables of the model, we will use a similar notation. It is important to point out that, at each time step *k*, only the first move of the input solution of the optimization problem, Δu(0|k), is implemented in the system. As a consequence, at each time step k≥1, $u(k)=\sum_{\ell =1}^{k}\Delta u(0|\ell )$,


$$\begin{array}{cc} x_{s}(0|k) & =x_{s}(0|k-1)+D_{0}\Delta u(0|k),\\ x_{d}(0|k) & =Fx_{d}(0|k-1)+D_{d}\Delta u(0|k),\\ y(0|k) & =x_{s}(0|k)+\Psi x_{d}(0|k), \end{array}$$

and, for j=1,…,m−1,


$$\begin{array}{cc} x_{s}(j|k) & =x_{s}(j-1|k)+D_{0}\Delta u(j|k),\\ x_{d}(j|k) & =Fx_{d}(j-1|k)+D_{d}\Delta u(j|k),\\ y(j|k) & =x_{s}(j|k)+\Psi x_{d}(j|k). \end{array}$$

The EIHMPC is based on the following cost function


$$V_{k}:=\sum_{j=0}^{\infty }[e(j|k)-\delta_{k}{]}^{T}Q[e(j|k)-\delta_{k}]+\sum_{j=0}^{m-1}\Delta u(j|k{)}^{T}R\Delta u(j|k)+\delta_{k}^{T}S\delta_{k},\;k\geq 1,$$

where e(j|k):=y(j|k)−r, $r\in {\mathbb{R}}^{n_{y}}$ is the set-point, $\delta_{k}\in {\mathbb{R}}^{n_{y}}$ is a *slack* vector, $Q\in {\mathbb{R}}^{n_{y}\times n_{y}}$, $R\in {\mathbb{R}}^{n_{u}\times n_{u}}$ and $S\in {\mathbb{R}}^{n_{y}\times n_{y}}$ are positive definite. To prevent the above cost from being unbounded, as discussed in [4], the following constraint is imposed


$$x_{s}(m-1|k)-\delta_{k}-r=0,\;k\geq 1.$$ (3)

We will work under the following assumption on the OPOM, which was already present in [4].

**Assumption 1.** *The system is stable, that is, the spectral radius of F is strictly smaller than 1.*

Using Assumption 1 and (3), we obtain, for k≥1,


$$\begin{array}{cc} V_{k} & =\sum_{j=0}^{m-1}[e(j|k)-\delta_{k}{]}^{T}Q[e(j|k)-\delta_{k}]+x_{d}(m-1|k{)}^{T}\bar{Q}x_{d}(m-1|k)\\ & +\sum_{j=0}^{m-1}\Delta u(j|k{)}^{T}R\Delta u(j|k)+\delta_{k}^{T}S\delta_{k}\\ & =\sum_{j=0}^{m-1}|e(j|k)-\delta_{k}{|}_{Q}^{2}+|x_{d}(m-1|k){|}_{\bar{Q}}^{2}+\sum_{j=0}^{m-1}|\Delta u(j|k){|}_{R}^{2}+|\delta_{k}{|}_{S}^{2}, \end{array}$$ (4)

where


$$\bar{Q}:=\sum_{j=1}^{\infty }(F^{j}{)}^{T}\Psi^{T}Q\Psi F^{j}$$

is positive semidefinite. Also, note that $\bar{Q}-F^{T}\bar{Q}F=F^{T}\Psi^{T}Q\Psi F$.

Now, in order to state the control optimization problem, we first fix 𝕌 and Δ𝕌 rectangles in ${\mathbb{R}}^{n_{u}}$ containing the origin **0**. Let us also define


$$\Delta u_{k}=[\Delta u(0|k{)}^{T}\;\Delta u(1|k{)}^{T}\;\cdots \;\Delta u(m-1|k{)}^{T}{]}^{T}\in {\mathbb{R}}^{mn_{u}}.$$

**Control Optimization problem**: The control optimization problem of the EIHMPC can be stated as follows: at each time step k≥1, we determine $\left(\Delta u_{k},\delta_{k}\right)$ that minimizes *V*_*k*_ subject to constraints (3),


$$u(k-1)+\sum_{j=0}^{i}\Delta u(j|k)\in 𝕌,\;\text{for}\;0\leq i<m,\;\text{and }\;\;\Delta u(j|k)\in \Delta 𝕌,\text{ for }0\leq j<m.$$

Without loss of generality, we assume that, at time 0, the system is in the steady state u(0)=0, $x_{s}(0)=0$, $x_{d}(0)=0$. Since 𝕌 and Δ𝕌 are convex sets and *R* is positive definite, the solution of the above optimization problem is unique. Let $\Delta u_{k}^{*}$, $\delta_{k}^{*}$ denote the solution at time step *k*, and let $V_{k}^{*}$ be the corresponding cost,


$$V_{k}^{*}=\sum_{j=0}^{m-1}|e^{*}(j|k)-\delta_{k}^{*}{|}_{Q}^{2}+|x_{d}^{*}(m-1|k){|}_{\bar{Q}}^{2}+\sum_{j=0}^{m-1}|\Delta u^{*}(j|k){|}_{R}^{2}+|\delta_{k}^{*}{|}_{S}^{2},$$

where $e^{*}$, $x_{s}^{*}$ and $x_{d}^{*}$ are obtained from $\Delta u_{k}^{*}$, (1) and (2).

We also need the following

**Assumption 2.** *The output reference r is such that* $r=D_{0}u_{r}$ *for some* $u_{r}\in 𝕌$*.*

We finally state the results about convergence and stability for the EIHMPC. We emphasize that we do not suppose that the gain matrix *D*_0_ is regular in the following theorems.

**Theorem 1** (Convergence). *Under Assumptions 1 and 2, we can choose the matrix S such that*


$$\lim_{k\to \infty }V_{k}^{*}=0.\;]$$

**Theorem 2** (Stability)*. Under Assumptions 1 and 2, the controller is stable, that is, for any* η>0*, there exists* ε>0 *such that* ‖r‖≤ε *implies that* $\|e^{*}(0|k)\|\leq \eta$ *for all* k≥1*.*

## Proofs of Theorems 1 and 2

The proof of Theorem 1 is based on the following key ideas:

1. Non-Increasing Nature of the Cost Function (Lemma 1): This result was already established in [4] and states that the optimal cost function $V_{k}^{*}$ is non-increasing in *k*.
2. Convergence of the Dynamic State (Lemma 2) It is shown that the dynamic part of the state *x*_*d*_ converges to zero as k→∞.
3. Convergence of the Orthogonal Component of the Input Sum (Lemma 3): This lemma proves that the orthogonal component of the sum of future control input increments tends to zero as k→∞. The result uses Lemmas 1 and 2, together with the terminal state constraint on *x*_*s*_. Here, the deviation $e_{s}(0|k)-\delta_{k}$ is expressed in terms of the OPOM matrix *D*_0_ and the sum of input increments. We also exploit the fact that the restriction of *D*_0_ to the orthogonal complement of its kernel, denoted $D_{0}^{⟂}$, is a linear isomorphism. This formulation emphasizes that *D*_0_ may be a singular matrix.
4. Convergence of the Control Input to the Reference Affine Subspace (Proposition 1): This proposition demonstrates that if the matrix *S* is correctly chosen, the distance between the optimal control input $u^{*}(0|k)$ and the affine subspace *U*_*r*_ (which contains *u*_*r*_ such that $D_{0}u_{r}=r$) converges to zero as k→∞.
5. Proof by Contradiction: Combining Proposition 1 and Lemmas 2 and 3, the final argument to prove Theorem 1 goes by contradiction, leading to the conclusion that $V_{k}^{*}\to 0$ as k→∞.

To prove Theorem 2, we propose a feasible initial solution with an associated cost such that ${\tilde{V}}_{1}=\|r{\|}_{S}^{2}$. Then, using the parallelogram law, the optimal output deviation is shown to be bounded from above (up to a constant) by the euclidean norm of *r* at each time step. Consequently, we conclude that if the set-point *r* is sufficiently close to the starting point of the process (which is located at the origin), the output deviation also remains small at each time step, thereby confirming nominal stability.

We start with several technical results that will be useful to prove Theorems 1 and 2. The following lemma was already established in [4], but for the sake of completeness we present its proof.

**Lemma 1.** *Under Assumption 1, the sequence* $(V_{k}^{*}{)}_{k\geq 1}$ *is non-increasing.*

*Proof.* At time step *k* + 1, let $\Delta {\tilde{u}}_{k+1},{\tilde{\delta }}_{k+1}$ be such that


$$\begin{array}{cc} \Delta \tilde{u}(j|k+1) & =\Delta u^{*}(j+1|k),\text{ for }j=0,1,\ldots ,m-2,\\ \Delta \tilde{u}(m-1|k+1) & =0,\\ {\tilde{\delta }}_{k+1} & =\delta_{k}^{*}. \end{array}$$

Note that $\Delta {\tilde{u}}_{k+1},{\tilde{\delta }}_{k+1}$ is a feasible strategy for the control optimization problem at time step *k* + 1 (by “feasible strategy” we mean that $\Delta {\tilde{u}}_{k+1},{\tilde{\delta }}_{k+1}$ satisfies all the constraints of the optimization problem). Indeed, by letting $e_{s}(j|k):=x_{s}(j|k)-r$, we have that


$$\begin{array}{cc} e_{s}^{*}(0|k)+D_{0}\sum_{j=0}^{m-1}\Delta \tilde{u}(j|k+1)-{\tilde{\delta }}_{k+1} & =e_{s}^{*}(0|k)+D_{0}\sum_{j=1}^{m-1}\Delta u^{*}(j|k)-\delta_{k}^{*}\\ & =e_{s}^{*}(0|k-1)+D_{0}\sum_{j=0}^{m-1}\Delta u^{*}(j|k)-\delta_{k}^{*}=0, \end{array}$$

so that (3) is satisfied. Moreover, note that $\Delta \tilde{u}(j|k+1)\in \Delta 𝕌$ for 0≤j≤m−1, and recalling that $\Delta u^{*}(m|k)=0$, we obtain


$$u^{*}(k)+\sum_{j=0}^{i}\Delta \tilde{u}(j|k+1)=u^{*}(k)+\sum_{j=1}^{i+1}\Delta u^{*}(j|k)=u^{*}(k-1)+\sum_{j=0}^{i+1}\Delta u^{*}(j|k)\in 𝕌$$

for 0≤i≤m−1.

The cost corresponding to this strategy is


$$\begin{array}{cc} {\tilde{V}}_{k+1} & =\sum_{j=0}^{m-1}|\tilde{e}(j|k+1)-{\tilde{\delta }}_{k+1}{|}_{Q}^{2}+|{\tilde{x}}_{d}(m-1|k+1){|}_{\bar{Q}}^{2}\\ & +\sum_{j=0}^{m-1}|\Delta \tilde{u}(j|k+1){|}_{R}^{2}+|{\tilde{\delta }}_{k+1}{|}_{S}^{2}\\ & =\sum_{j=0}^{m-1}|\tilde{e}(j|k+1)-\delta_{k}^{*}{|}_{Q}^{2}+|{\tilde{x}}_{d}(m-1|k+1){|}_{\bar{Q}}^{2}+\sum_{j=1}^{m-1}|\Delta u^{*}(j|k){|}_{R}^{2}+|\delta_{k}^{*}{|}_{S}^{2} \end{array}$$

and, since $\tilde{e}(j|k+1)=e^{*}(j+1|k)$ for 0≤j≤m−2, we have that


$${\tilde{V}}_{k+1}-V_{k}^{*}=|\tilde{e}(m-1|k+1)-\delta_{k}^{*}{|}_{Q}^{2}-|e^{*}(0|k)-\delta_{k}^{*}{|}_{Q}^{2}+|{\tilde{x}}_{d}(m-1|k+1){|}_{\bar{Q}}^{2}\;-|x_{d}^{*}(m-1|k){|}_{\bar{Q}}^{2}-|\Delta u^{*}(0|k){|}_{R}^{2}.$$

Now observe that


$$\tilde{e}(m-1|k+1)-\delta_{k}^{*}={\tilde{e}}_{s}(m-1|k+1)+\Psi {\tilde{x}}_{d}(m-1|k+1)-\delta_{k}^{*}=\Psi {\tilde{x}}_{d}(m-1|k+1),$$

$${\tilde{x}}_{d}(m-1|k+1)=F{\tilde{x}}_{d}(m-2|k+1)+D_{d}\Delta \tilde{u}(m-1|k+1)=F{\tilde{x}}_{d}(m-2|k+1)$$

and


$$\begin{array}{cc} {\tilde{x}}_{d}(m-2|k+1) & =F^{m-1}x_{d}^{*}(0|k)+\sum_{i=0}^{m-2}F^{m-2-i}D_{d}\Delta \tilde{u}(i|k+1)\\ & =F^{m-1}x_{d}^{*}(0|k)+\sum_{i=1}^{m-1}F^{m-1-i}D_{d}\Delta u^{*}(i|k)\\ & =x_{d}^{*}(m-1|k), \end{array}$$

so that


$$\begin{array}{cc} & |\tilde{e}(m-1|k+1)-\delta_{k}^{*}{|}_{Q}^{2}+|{\tilde{x}}_{d}(m-1|k+1){|}_{\bar{Q}}^{2}\\ & \;=|\Psi Fx_{d}^{*}(m-1|k){|}_{Q}^{2}+|Fx_{d}^{*}(m-1|k){|}_{\bar{Q}}^{2}\\ & \;=|x_{d}^{*}(m-1|k){|}_{(\Psi F{)}^{T}Q(\Psi F)+F^{T}\bar{Q}F}^{2}\\ & \;=|x_{d}^{*}(m-1|k){|}_{\bar{Q}}^{2} \end{array}$$

and then


$${\tilde{V}}_{k+1}-V_{k}^{*}=-|e^{*}(0|k)-\delta_{k}^{*}{|}_{Q}^{2}-|\Delta u^{*}(0|k){|}_{R}^{2}\leq 0.$$

Since the strategy $\Delta {\tilde{u}}_{k+1},{\tilde{\delta }}_{k+1}$ at time step *k* + 1 is not necessarily the optimal one, we have that $V_{k+1}^{*}\leq {\tilde{V}}_{k+1}$ and the sequence $(V_{k}^{*}{)}_{k\geq 1}$ is non-increasing. □

**Lemma 2.** *Under Assumption 1, we have that*


$$x_{d}^{*}(0|k)\to 0,\;as\;k\to \infty .$$

*Proof.* From the proof of Lemma 1, we have that


$$V_{k+1}^{*}-V_{k}^{*}\leq -|e^{*}(0|k)-\delta_{k}^{*}{|}_{Q}^{2}-|\Delta u^{*}(0|k){|}_{R}^{2}.$$

Observe that $(V_{k}^{*}{)}_{k\geq 1}$ converges since it is non-increasing and non-negative. Therefore we deduce that


$$|e^{*}(0|k)-\delta_{k}^{*}{|}_{Q}^{2}\overset{k\to \infty \;}{\to }0\text{ and }|\Delta u^{*}(0|k){|}_{R}^{2}\overset{k\to \infty \;}{\to }0.$$ (5)

Moreover, for p≥1, we have that


$$x_{d}^{*}(0|k+p)=F^{p}x_{d}^{*}(0|k)+\sum_{j=0}^{p-1}F^{j}D_{d}\Delta u^{*}(0|k+p-j).$$

Now, let ‖·‖ be the euclidean norm on ${\mathbb{R}}^{n_{d}}$. By Assumption 1, we have that


$$\sum_{j=0}^{\infty }\|F^{j}\|<\infty .$$

Hence, for any ε>0, there exists $k_{0}\geq 1$ such that


$$\sup_{p\geq 1}|x_{d}^{*}(0|k_{0}+p)-F^{p}x_{d}^{*}(0|k_{0})|\leq \sup_{j\geq 1}|D_{d}\Delta u^{*}(0|k_{0}+j)|\sum_{j=0}^{\infty }\|F^{j}\|<\varepsilon /2,$$

and, since $\lim_{j\to \infty }F^{j}=0$, there exists $p_{0}\geq 1$ such that, for all $p\geq p_{0}$,


$$|F^{p}x_{d}^{*}(0|k_{0})|<\varepsilon /2.$$

Finally, for all $p\geq p_{0}$,


$$|x_{d}^{*}(0|k_{0}+p)|\leq |F^{p}x_{d}^{*}(0|k_{0})|+\sup_{p\geq 1}|x_{d}^{*}(0|k_{0}+p)-F^{p}x_{d}^{*}(0|k_{0})|<\varepsilon ,$$

that is, $x_{d}^{*}(0|k)\to 0$ as k→∞. □

We now equip ${\mathbb{R}}^{n_{u}}$ with the euclidean norm ‖·‖ and the associated scalar product ⟨·,·⟩. We decompose


$${\mathbb{R}}^{n_{u}}=\ker D_{0}⊕(\ker D_{0}{)}^{⟂}.$$

We also denote by $D_{0}^{⟂}$ the restriction of *D*_0_ to $(\ker D_{0}{)}^{⟂}$ with values in $\text{Im}D_{0}\subset {\mathbb{R}}^{n_{y}}$. Observe that $D_{0}^{⟂}$ is a linear isomorphism. For a vector $x\in {\mathbb{R}}^{n_{u}}$, we denote by $x_{⟂}$ the unique vector in $(\ker D_{0}{)}^{⟂}$ such that $x-x_{⟂}\in \ker D_{0}$.

**Lemma 3.** *Under Assumption 1 we have that*


$$(\sum_{j=0}^{m-1}\Delta u^{*}(j|k){)}_{⟂}\to 0,\;as\;k\to \infty .$$

*Proof.* Recall that $e_{s}(j|k):=x_{s}(j|k)-r$. Since


$$e^{*}(0|k)=e_{s}^{*}(0|k)+\Psi x_{d}^{*}(0|k)$$

and by Lemma 2


$$e^{*}(0|k)-\delta_{k}^{*}\overset{k\to \infty \;}{\to }0\text{ and }x_{d}^{*}(0|k)\overset{k\to \infty \;}{\to }0,$$

we have that


$$e_{s}^{*}(0|k)-\delta_{k}^{*}\overset{k\to \infty \;}{\to }0.$$

But, using (3), we have that


$$e_{s}^{*}(0|k)-\delta_{k}^{*}=-D_{0}(\sum_{j=1}^{m-1}\Delta u^{*}(j|k){)}_{⟂}=-D_{0}^{⟂}(\sum_{j=1}^{m-1}\Delta u^{*}(j|k){)}_{⟂}$$

so that, since $D_{0}^{⟂}$ is a linear isomorphism,


$$(\sum_{j=1}^{m-1}\Delta u^{*}(j|k){)}_{⟂}\overset{k\to \infty \;}{\to }0$$

and finally, by (5), we obtain


$$(\sum_{j=0}^{m-1}\Delta u^{*}(j|k){)}_{⟂}\overset{k\to \infty \;}{\to }0.$$

Recall Assumption 2 and let us denote by $U_{r}:=u_{r}+\ker D_{0}$. Observe that for any $u\in U_{r}$, *D_0_u*=*r*. Let us also denote by *P*_*r*_ the orthogonal projection on the affine subspace *U*_*r*_. We recall that *P*_*r*_ is not a linear map unless r=0.

We now build up the matrix *S* (in the canonical basis of ${\mathbb{R}}^{n_{y}}$) that will be considered later on. For this, fix respectively an orthonormal basis of $(\ker D_{0}{)}^{⟂}$, $\left\{v_{1},v_{2},\ldots ,v_{k}\right\}$, an orthonormal basis of Im *D*_0_, $\left\{w_{1},w_{2},\ldots ,w_{k}\right\}$, and an orthonormal basis of $(\text{Im}D_{0}{)}^{⟂}$, $\left\{w_{k+1},w_{k+2},\ldots ,w_{n_{y}}\right\}$. Consider the square matrix *M* of $D_{0}^{⟂}$ in the first two basis. Using the *LQ*-factorization, there exists a lower triangular matrix *L* and an orthogonal matrix *O* such that *M* = *LO*. Since *M* is regular, we have that *L* is regular. Now, consider the matrix *K*_1_ (resp. *K*_2_) of dimension $k\times n_{y}$ (resp. $(n_{y}-k)\times n_{y}$) whose column vectors are the orthogonal projections of the canonical vectors of ${\mathbb{R}}^{n_{y}}$ on $\left\{w_{1},w_{2},\ldots ,w_{k}\right\}$ (resp. $\left\{w_{k+1},w_{k+2},\ldots ,w_{n_{y}}\right\}$). Define $\hat{S}=K_{1}^{T}(L^{-1}{)}^{T}L^{-1}K_{1}+K_{2}^{T}K_{2}$, which is positive definite on ${\mathbb{R}}^{n_{y}}$. Furthermore, we can check that for all $v\in (\ker D_{0}{)}^{⟂}$, $\|D_{0}v{\|}_{\hat{S}}^{2}=(D_{0}v{)}^{T}\hat{S}D_{0}v=\|v{\|}^{2}$.

**Proposition 1.** *Consider* $S=\beta \hat{S}$ *with* β *a positive real number. We can choose* β *large enough (depending only on the parameters of the model other than S) such that under Assumptions 1 and 2 we have that*


$${lim sup}_{k\to \infty }\|u^{*}(0|k)-P_{r}u^{*}(0|k)\|=0.$$

*Proof.* The proof goes by contradiction, that is, assume that ${lim sup}_{k\to \infty }\|u^{*}(0|k)-P_{r}u^{*}(0|k)\|=c>0$. Then, for all ε>0 there exists a subsequence $(k_{n}{)}_{n\geq 1}$ such that for all n≥1,


$$\|u^{*}(0|k_{n}-1)-P_{r}u^{*}(0|k_{n}-1)\|\geq c-\frac{\varepsilon }{2}.$$

By Lemma 3, there exists *n*_0_ such that, for $n\geq n_{0}$,


$$\|(u^{*}(m-1|k_{n})-u^{*}(0|k_{n}-1){)}_{⟂}\|\leq \frac{\varepsilon }{2}.$$

This implies that for $n\geq n_{0}$,


$$\|u^{*}(m-1|k_{n})-P_{r}u^{*}(m-1|k_{n})\|\geq c-\varepsilon .$$ (6)

Indeed, applying the triangle inequality and using the fact that $(P_{r}v-P_{r}w{)}_{⟂}=0$ for all $v,w\in {\mathbb{R}}^{n_{u}}$, we obtain


$$\begin{array}{cc} \left\|u^{*}(m-1|k_{n})-P_{r}u^{*}(m-1|k_{n})\right\| & =\|(u^{*}(m-1|k_{n})-P_{r}u^{*}(m-1|k_{n}){)}_{⟂}\|\\ & \geq \|(u^{*}(0|k_{n}-1)-P_{r}u^{*}(m-1|k_{n}){)}_{⟂}\|\\ & -\|(u^{*}(m-1|k_{n})-u^{*}(0|k_{n}-1){)}_{⟂}\|\\ & =\|u^{*}(0|k_{n}-1)-P_{r}u^{*}(0|k_{n}-1)\|\\ & -\|(u^{*}(m-1|k_{n})-u^{*}(0|k_{n}-1){)}_{⟂}\|\\ & \geq c-\frac{\varepsilon }{2}-\frac{\varepsilon }{2}\\ & =c-\varepsilon . \end{array}$$

Also, there exists *k*_0_ such that for all $k\geq k_{0}$ we have


$$\|u^{*}(0|k-1)-P_{r}u^{*}(0|k-1)\|\leq c+\varepsilon .$$ (7)

Then for some α∈(0,1] to be chosen later, consider the following strategy at time *k* such that $k\geq k_{0}$ and *k* = *k*_*n*_ for some $n\geq n_{0}$.


$$\Delta \tilde{u}(0|k)=\alpha (\Pi_{r}(u^{*}(0|k-1))-u^{*}(0|k-1))$$

where $\Pi_{r}x$ is the point in $𝕌\cap U_{r}$ such that $\left\|x-\Pi_{r}x\|=\text{dist}(x,𝕌\cap U_{r}\right)$, $\Delta \tilde{u}(j|k)=0$ for j≥1 and ${\tilde{\delta }}_{k}$ given by


$${\tilde{\delta }}_{k}=e_{s}^{*}(0|k-1)+D_{0}\Delta \tilde{u}(0|k).$$

Since 𝕌 is convex, we can choose α small enough such that this strategy is indeed feasible. We now compute the cost of this strategy and compare it to the optimal cost. First, observe that


$$\begin{array}{cc} {\tilde{\delta }}_{k} & =e_{s}^{*}(0|k-1)+D_{0}\Delta \tilde{u}(0|k)\\ & =D_{0}u^{*}(0|k-1)-r+\alpha D_{0}[\Pi_{r}u^{*}(0|k-1)-u^{*}(0|k-1)]\\ & =(1-\alpha )D_{0}u^{*}(0|k-1)-r+\alpha r\\ & =(1-\alpha )(D_{0}u^{*}(0|k-1)-r)\\ & =(1-\alpha )D_{0}[u^{*}(0|k-1)-\Pi_{r}u^{*}(0|k-1)]. \end{array}$$

Thus, using (7), we obtain that


$$\begin{array}{cc} \|{\tilde{\delta }}_{k}{\|}_{S}^{2} & =(1-\alpha {)}^{2}\|D_{0}[u^{*}(0|k-1)-\Pi_{r}u^{*}(0|k-1)]{\|}_{S}^{2}\\ & =(1-\alpha {)}^{2}\|D_{0}[u^{*}(0|k-1)-\Pi_{r}u^{*}(0|k-1){]}_{⟂}{\|}_{S}^{2}\\ & =(1-\alpha {)}^{2}\beta \|[u^{*}(0|k-1)-\Pi_{r}u^{*}(0|k-1){]}_{⟂}{\|}^{2}\\ & =(1-\alpha {)}^{2}\beta \|u^{*}(0|k-1)-P_{r}u^{*}(0|k-1){\|}^{2}\\ & \leq (1-\alpha {)}^{2}\beta (c+\varepsilon {)}^{2}. \end{array}$$

On the other hand, we have


$$\begin{array}{cc} \delta_{k}^{*} & =e_{s}^{*}(0|k-1)+D_{0}\sum_{j=0}^{m-1}\Delta u^{*}(j|k)\\ & =D_{0}u^{*}(m-1|k)-r\\ & =D_{0}[u^{*}(m-1|k)-\Pi_{r}u^{*}(m-1|k){]}_{⟂}. \end{array}$$

Hence, using (6), we obtain that


$$\begin{array}{cc} \|\delta_{k}^{*}{\|}_{S}^{2} & =\|D_{0}[u^{*}(m-1|k)-\Pi_{r}u^{*}(m-1|k){]}_{⟂}{\|}_{S}^{2}\\ & =\beta \|[u^{*}(m-1|k)-\Pi_{r}u^{*}(m-1|k){]}_{⟂}{\|}^{2}\\ & =\beta \|u^{*}(m-1|k)-P_{r}u^{*}(m-1|k){\|}^{2}\\ & \geq \beta (c-\varepsilon {)}^{2}. \end{array}$$

After some elementary computation, we obtain that


Vk*−V~k≥‖δk*‖S2−‖δ~k‖S2−‖Δu~(0|k)‖Z2**−2(∑j=0m−1⟨ΨFj+1xd*(0|k−1),ΨFjDdΔu~(0|k)⟩Q          +⟨Fmxd*(0|k−1),Fm−1DdΔu~(0|k)⟩Q¯)

where


$$\begin{array}{cc} Z & =R+\sum_{j=0}^{m-1}(D_{d}{)}^{T}(F^{j}{)}^{T}\Psi^{T}Q\Psi F^{j}D_{d}+D_{d}^{T}(F^{m-1}{)}^{T}\bar{Q}F^{m-1}D_{d}\\ & =:R+D_{d}^{T}GD_{d}. \end{array}$$ (8)

Using the Cauchy-Schwarz inequality, we have that


Vk*−V~k≥‖δk*‖S2−‖δ~k‖S2−‖Δu~(0|k)‖Z2**−2(∑j=0m−1‖ΨFj+1xd*(0|k−1)‖Q‖ΨFjDdΔu~(0|k)‖Q+‖Fmxd*(0|k−1)‖Q¯‖Fm−1DdΔu~(0|k)‖Q¯)≥‖δk*‖S2−‖δ~k‖S2−‖Δu~(0|k)‖Z2−C1‖xd*(0|k−1)‖‖Δu~(0|k)‖ (9)

for some positive constant *C*_1_ that depends on $m,\Psi ,F,D_{d}$ and *Q*. Thus, we obtain that


$$V_{k}^{*}-{\tilde{V}}_{k}\geq \|\delta_{k}^{*}{\|}_{S}^{2}-\|{\tilde{\delta }}_{k}{\|}_{S}^{2}-C_{2}\|\Delta \tilde{u}(0|k)\|(\|\Delta \tilde{u}(0|k)\|+\|x_{d}^{*}(0|k-1)\|)$$

for some positive constant *C*_2_ that depends on $m,R,\Psi ,F,D_{d}$ and *Q*.

Now let $\theta_{x}$ be the angle between $P_{r}x-x$ and $\Pi_{r}x-x$ for $x\in 𝕌⧵U_{r}$. Since 𝕌 is a rectangle we have that $\varphi :=\inf_{x\in 𝕌⧵U_{r}}|\cos\theta_{x}|>0$ and therefore $\left\|\Delta \tilde{u}(0|k)\|\leq \alpha \varphi^{-1}(c+\varepsilon \right)$. Now using Lemma 2, we can choose *k* large enough such that $\left\|x_{d}^{*}(0|k-1)\|\leq \alpha \varphi^{-1}(c+\varepsilon \right)$. Therefore, we obtain,


$$\begin{array}{cc} V_{k}^{*}-{\tilde{V}}_{k} & \geq \|\delta_{k}^{*}{\|}_{S}^{2}-\|{\tilde{\delta }}_{k}{\|}_{S}^{2}-C_{3}\alpha^{2}(c+\varepsilon {)}^{2}\\ & \geq \beta (c-\varepsilon {)}^{2}-(1-\alpha {)}^{2}\beta (c+\varepsilon {)}^{2}-C_{3}\alpha^{2}(c+\varepsilon {)}^{2} \end{array}$$

for some positive constant *C*_3_ (see the Appendix for an explicit expression) that depends on the parameters of the model other than *S*. Now, let us take ε<c/3 small enough and α=3ε/(c+ε) such that the strategy given by $\Delta \tilde{u}(0|k)$ is feasible. Consider $\beta >6C_{3}$. We can check that in this case $V_{k}^{*}-{\tilde{V}}_{k}>0$, which gives us the desired contradiction. □

### Proof of Theorem 1

From Proposition 1 and Lemma 3, we deduce that $\lim_{k\to \infty }\|\delta_{k}^{*}{\|}_{S}=0$. Indeed, recalling that $\|D_{0}v{\|}_{\hat{S}}^{2}=\|v{\|}^{2}$ for all $v\in (\ker D_{0}{)}^{⟂}$, observe that


$$\begin{array}{cc} \|\delta_{k}^{*}{\|}_{S} & \leq \|D_{0}[u^{*}(0|k-1)-P_{r}u^{*}(0|k-1)]{\|}_{S}+|D_{0}\sum_{j=0}^{m-1}\Delta u^{*}(j|k){|}_{S}\\ & =\sqrt{\beta }\|u^{*}(0|k-1)-P_{r}u^{*}(0|k-1)\|+\sqrt{\beta }|(\sum_{j=0}^{m-1}\Delta u^{*}(j|k){)}_{⟂}|\\ & \to 0+0 \end{array}$$

as k→∞.

Finally, we will show that $\lim_{k\to \infty }V_{k}^{*}=0$. To this end, we first observe that using (4), since $\lim_{k\to \infty }\|\delta_{k}^{*}{\|}_{S}=0$, if ${lim sup}_{k\to \infty }\sum_{j=0}^{m-1}\|\Delta u^{*}(j|k){\|}_{R}^{2}=0$ then $\lim_{k\to \infty }V_{k}^{*}=0$. Thus, assume that $\lim_{k\to \infty }V_{k}^{*}>0$. This implies that ${lim sup}_{k\to \infty }\sum_{j=0}^{m-1}\|\Delta u^{*}(j|k){\|}_{R}^{2}=c^{*}>0$ and therefore there exists some subsequence $(k_{n}{)}_{n\geq 1}$ such that for all n≥1, $\sum_{j=0}^{m-1}\|\Delta u^{*}(j|k){\|}_{R}^{2}\geq c^{*}/2$. Now consider $\varepsilon <c^{*}/2$. For *n* large enough using the strategy $\Delta \tilde{u}(j|k_{n})=0$, for all 0≤j<m and ${\tilde{\delta }}_{k_{n}}=\delta_{k_{n}}^{*}-D_{0}\sum_{j=0}^{m-1}\Delta u^{*}(j|k_{n})$ (we can check that it is indeed feasible) we obtain by Lemmas 2 and 3


$$\begin{array}{cc} {\tilde{V}}_{k_{n}} & =\|Fx_{d}^{*}(0|k_{n}-1){\|}_{G}^{2}+\|{\tilde{\delta }}_{k_{n}}{\|}_{S}^{2}\\ & \leq \|Fx_{d}^{*}(0|k_{n}-1){\|}_{G}^{2}+(|D_{0}\sum_{j=0}^{m-1}\Delta u^{*}(j|k_{n}){|}_{S}+\|\delta_{k_{n}}^{*}{\|}_{S}{)}^{2}\\ & \leq \varepsilon \\ & <c^{*}/2\\ & \leq V_{k_{n}}^{*} \end{array}$$

where *G* is from (8). This gives us the desired contradiction. □

### Proof of Theorem 2

Recall that the system is assumed to be initially in the steady state u(0)=0, $x_{s}(0)=0$, $x_{d}(0)=0$, and consider the following feasible strategy for the control optimization problem at time step *k* = 1,


$$\begin{array}{cc} \Delta \tilde{u}(j|1) & =0,\text{ for }j=0,1,\ldots ,m-1,\\ {\tilde{\delta }}_{1} & =-r, \end{array}$$

which has an associated cost ${\tilde{V}}_{1}=\|r{\|}_{S}^{2}$. Since the above strategy is not necessarily the optimal one, and using Lemma 1, we have that $V_{k}^{*}\leq V_{1}^{*}\leq {\tilde{V}}_{1}$ for all k≥1. But, for any k≥1,


$$\|e^{*}(0|k)-\delta_{k}^{*}{\|}_{Q}^{2}+\|\delta_{k}^{*}{\|}_{S}^{2}\leq V_{k}^{*}$$

and, on the other hand, by the parallelogram law,


$$\left\|e^{*}(0|k){\|}_{Q}^{2}\leq 2(\|e^{*}(0|k)-\delta_{k}^{*}{\|}_{Q}^{2}+\|\delta_{k}^{*}{\|}_{Q}^{2})\leq C_{1}(\|e^{*}(0|k)-\delta_{k}^{*}{\|}_{Q}^{2}+\|\delta_{k}^{*}{\|}_{S}^{2}\right)$$

for some positive constant *C*_1_. Thus we have that $\left\|e^{*}(0|k)\|\leq C_{2}\|r\right\|$ for some positive constant *C*_2_, for all k≥1. This implies that, for any η>0, there exists ε>0 such that $\|e^{*}(0|k)\|\leq \eta$ for all k≥1, if ‖r‖≤ε. □

### Extended Infinite Horizon MPC with zone control

In this section, we present the EIHMPC with zone control. The Formulation and results section introduces the controller formulation, which is based on the OPOM described previously in the Extended Infinite Horizon MPC section. This MPC formulation has two key features: the first one is that the set-point *r* is treated as an optimization variable; therefore, we denote it here as *y*_*sp*_. The second feature is that the controller receives external input targets *u*_*des*_.

We begin by defining the cost function and the terminal constraints: a terminal state constraint on *x*_*s*_, and an input constraint that links the control inputs to the external target *u*_*des*_. At each time step *k*, the optimization variables of this MPC are the sequence of input increments $\Delta u_{k}$, the output reference vector *y*_*sp*,*k*_, the slack variables associated with the terminal state constraint $\delta_{y,k}$, and those slacks related to the input constraint $\delta_{u,k}$.

For this controller, we work under Assumption 1 (which for convenience will be renamed as Assumption 3 below) and Assumption 4, which states that the input target *u*_*des*_ is reachable, i.e., belongs to the input domain 𝕌, and the corresponding predicted steady state $D_{0}u_{des}$ belongs to the set-point domain 𝕐. Assumption 4 guarantees the consistency between the input target and the set-point domain. In practice, without this assumption, the controller will not be able to guide, at the same time, the input to its target and the output to the set-point.

Based on these assumptions, Theorems 3 and 4 establish the convergence and stability of the closed-loop system under the EIHMPC with zone control.

### Formulation and results

In this section, we consider the EIHMPC with zone control [5]. This controller uses the OPOM given by (1) and (2) for prediction. The input target $u_{des}\in {\mathbb{R}}^{n_{u}}$ is sent at each time step by the Real Time Optimization (RTO) layer to the MPC layer. Unlike [4], in this MPC the set-point $y_{sp}\in {\mathbb{R}}^{n_{y}}$ is a variable of the optimization problem and is calculated at each time step. Here, the exact values of the set-point are not important, as long as they remain inside a range with specified limits. As in the Extended Infinite Horizon MPC section, for j=0,1,…,m−1, we define Δu(j|k) as the *j*-th move of the input solution of the optimization problem (to be defined below) at time step *k*. For the other variables of the model, we will use a similar notation.

The EIHMPC with zone control is based on the following cost function: for k≥1,


$$\begin{array}{c} {𝒱}_{k}:=\sum_{j=0}^{\infty }[y(j|k)-y_{sp,k}-\delta_{y,k}{]}^{T}Q_{y}[y(j|k)-y_{sp,k}-\delta_{y,k}]\\ +\sum_{j=0}^{\infty }[u(j|k)-u_{des}-\delta_{u,k}{]}^{T}Q_{u}[u(j|k)-u_{des}-\delta_{u,k}]\\ +\sum_{j=0}^{m-1}\Delta u(j|k{)}^{T}R\Delta u(j|k)+\delta_{y,k}^{T}S_{y}\delta_{y,k}+\delta_{u,k}^{T}S_{u}\delta_{u,k}, \end{array}$$

where $\delta_{y,k}\in {\mathbb{R}}^{n_{y}}$ and $\delta_{u,k}\in {\mathbb{R}}^{n_{u}}$ are *slack* vectors, and $Q_{y}\in {\mathbb{R}}^{n_{y}\times n_{y}}$, $Q_{u}\in {\mathbb{R}}^{n_{u}\times n_{u}}$, $R\in {\mathbb{R}}^{n_{u}\times n_{u}}$, $S_{y}\in {\mathbb{R}}^{n_{y}\times n_{y}}$ and $S_{u}\in {\mathbb{R}}^{n_{u}\times n_{u}}$ are positive definite weighting matrices. To prevent the cost from being unbounded, as discussed in [5], we impose the terminal constraints


$$x_{s}(m-1|k)-y_{sp,k}-\delta_{y,k}=0,\;k\geq 1,$$ (10)

and


$$u(m-1|k)-u_{des}-\delta_{u,k}=0,\;k\geq 1.$$ (11)

In this section we also work under Assumption 1, which for convenience we rename as

**Assumption 3.** *The system is stable, that is, the spectral radius of the matrix F is strictly smaller than 1.*

Under Assumption 3 and constraints (10) and (11) we have that, for k≥1,


$$\begin{array}{c} {𝒱}_{k}=\sum_{j=0}^{m-1}[y(j|k)-y_{sp,k}-\delta_{y,k}{]}^{T}Q_{y}[y(j|k)-y_{sp,k}-\delta_{y,k}]+x_{d}(m-1|k{)}^{T}\bar{Q}x_{d}(m-1|k)\\ +\sum_{j=0}^{m-1}[u(j|k)-u_{des}-\delta_{u,k}{]}^{T}Q_{u}[u(j|k)-u_{des}-\delta_{u,k}]+\sum_{j=0}^{m-1}\Delta u(j|k{)}^{T}R\Delta u(j|k)\\ +\;\delta_{y,k}^{T}S_{y}\delta_{y,k}+\delta_{u,k}^{T}S_{u}\delta_{u,k} \end{array}$$

or, in other words,


$$\begin{array}{c} {𝒱}_{k}=\sum_{j=0}^{m-1}|y(j|k)-y_{sp,k}-\delta_{y,k}{|}_{Q_{y}}^{2}+|x_{d}(m-1|k){|}_{{\bar{Q}}_{y}}^{2}+\sum_{j=0}^{m-1}|u(j|k)-u_{des}-\delta_{u,k}{|}_{Q_{u}}^{2}\\ +\sum_{j=0}^{m-1}|\Delta u(j|k){|}_{R}^{2}+|\delta_{y,k}{|}_{S_{y}}^{2}+|\delta_{u,k}{|}_{S_{u}}^{2} \end{array}$$

where


$${\bar{Q}}_{y}:=\sum_{j=1}^{\infty }(F^{j}{)}^{T}\Psi^{T}Q_{y}\Psi F^{j}$$

is positive semidefinite and satisfies ${\bar{Q}}_{y}-F^{T}{\bar{Q}}_{y}F=F^{T}\Psi^{T}Q_{y}\Psi F$.

In order to state the control optimization problem, we first fix 𝕌 and Δ𝕌 rectangles in ${\mathbb{R}}^{n_{u}}$ and 𝕐 rectangle in ${\mathbb{R}}^{n_{y}}$, all of them containing the corresponding origin. Let us recall that


$$\Delta u_{k}=[\Delta u(0|k{)}^{T}\;\Delta u(1|k{)}^{T}\;\cdots \;\Delta u(m-1|k{)}^{T}{]}^{T}\in {\mathbb{R}}^{mn_{u}}.$$

**Control optimization problem**: The control optimization problem of the EIHMPC with zone control is the following: at each time step k≥1, we determine $\left(\Delta u_{k},y_{sp,k},\delta_{y,k},\delta_{u,k}\right)$ that minimizes ${𝒱}_{k}$ subject to constraints (10), (11),


$$u(k-1)+\sum_{j=0}^{i}\Delta u(j|k)\in 𝕌,\text{ for }0\leq i<m,\;\Delta u(j|k)\in \Delta 𝕌,\text{ for }0\leq j<m,\;\text{and}\;\;\;\;y_{sp,k}\in 𝕐.$$

Here we assume that, at time 0, the system is in the steady state *u*(0) = **0**, *x*_*s*_(0) = **0**, *x*_*d*_(0) = **0**. Since 𝕌, Δ𝕌 and 𝕐 are convex sets and *R* and *S*_*y*_ are positive definite, the solution of the above optimization problem is unique. Let $\Delta u_{k}^{*}$, $y_{sp,k}^{*}$, $\delta_{y,k}^{*}$, $\delta_{u,k}^{*}$ be the solution of the control optimization problem at time step *k* and let ${𝒱}_{k}^{*}$ be the corresponding cost,


$$\begin{array}{c} {𝒱}_{k}^{*}=\sum_{j=0}^{m-1}|y^{*}(j|k)-y_{sp,k}^{*}-\delta_{y,k}^{*}{|}_{Q_{y}}^{2}+|x_{d}^{*}(m-1|k){|}_{{\bar{Q}}_{y}}^{2}+\sum_{j=0}^{m-1}|u^{*}(j|k)-u_{des}-\delta_{u,k}^{*}{|}_{Q_{u}}^{2}\\ +\sum_{j=0}^{m-1}|\Delta u^{*}(j|k){|}_{R}^{2}+|\delta_{y,k}^{*}{|}_{S_{y}}^{2}+|\delta_{u,k}^{*}{|}_{S_{u}}^{2}, \end{array}$$

where $u^{*}$ is obtained from $\Delta u_{k}^{*}$, and $y^{*}$, $x_{s}^{*}$ and $x_{d}^{*}$ are obtained from $\Delta u_{k}^{*}$, (1) and (2).

In the following, we also need the

**Assumption 4.** *The input target u*_*des*_ *is such that* $u_{des}\in 𝕌$ *and* $D_{0}u_{des}\in 𝕐$*.*

We finally state our results about convergence and stability of this second controller.

**Theorem 3** (Convergence). *Let* $I_{n_{u}}$ *be the identity matrix of dimension n*_*u*_ *and H be the matrix given by*


$$H=(m-1)D_{0}^{T}Q_{y}D_{0}+(\Psi D_{d}{)}^{T}Q_{y}(\Psi D_{d})+D_{d}^{T}{\bar{Q}}_{y}D_{d}+(m-1)Q_{u}+R.$$

*Under Assumptions 3 and 4, if* $S_{u}>H+I_{n_{u}}$ *then*


$$\lim_{k\to \infty }{𝒱}_{k}^{*}=0.$$

**Theorem 4** (Stability). *Under Assumptions 3 and 4, the controller is stable, that is, for any* η>0*, there exists* ε>0 *such that* $\|u_{des}\|\leq \varepsilon$ *implies that*


$$|[\begin{array}{c} y^{*}(0|k)-y_{sp,k}^{*}\\ u^{*}(0|k)-u_{des} \end{array}]|\leq \eta \;for\;all\;k\geq 1.$$

### Proofs of Theorems 3 and 4

The proof of Theorem 3 follows a similar reasoning to that of Theorem 1, although it is simplified by the presence of the target *u*_*des*_ for the input variable *u*. Indeed, while in Lemma 3 we only have that


$$(\sum_{j=0}^{m-1}\Delta u^{*}(j|k){)}_{⟂}\to 0,\;\text{ as }k\to \infty ,$$

now the presence of the input target *u*_*des*_ allows us to prove that


$$\sum_{j=0}^{m-1}\Delta u^{*}(j|k)\to 0,\;\text{ as }k\to \infty ,$$

see Lemma 5. As a consequence, we obtain the apparently stronger fact that


$$\sum_{j=0}^{m-1}|\Delta u^{*}(j|k){|}_{R}^{2}\to 0,\;\text{ as }k\to \infty ,$$

see Corollary 1. This crucial fact enables us to prove by contradiction that ${𝒱}_{k}^{*}\to 0$ as k→∞, for a well chosen *S*_*u*_. The proof of Theorem 4 parallels the proof of Theorem 2.

We start with several technical results that will be useful to prove Theorems 3 and 4. The following lemma was established in [5], but for the sake of completeness we present its proof.

**Lemma 4.** *Under Assumption 3, the sequence* $({𝒱}_{k}^{*}{)}_{k\geq 1}$ *is non-increasing.*

*Proof.* Let $\Delta {\tilde{u}}_{k+1}$, ${\tilde{y}}_{sp,k+1}$, ${\tilde{\delta }}_{y,k+1}$, ${\tilde{\delta }}_{u,k+1}$ be the following strategy for the control optimization problem at time step *k* + 1:


$$\begin{array}{cc} \Delta \tilde{u}(j|k+1) & =\Delta u^{*}(j+1|k),\text{ for }j=0,1,\ldots ,m-2,\\ \Delta \tilde{u}(m-1|k+1) & =0,\\ {\tilde{y}}_{sp,k+1} & =y_{sp,k}^{*},\\ {\tilde{\delta }}_{y,k+1} & =\delta_{y,k}^{*},\\ {\tilde{\delta }}_{u,k+1} & =\delta_{u,k}^{*}. \end{array}$$

We can check that the above strategy is feasible for the control optimization problem at time step *k* + 1, and its cost is


$$\begin{array}{cc} {\tilde{𝒱}}_{k+1} & =\sum_{j=0}^{m-1}|\tilde{y}(j|k+1)-y_{sp,k}^{*}-\delta_{y,k}^{*}{|}_{Q_{y}}^{2}+|{\tilde{x}}_{d}(m-1|k+1){|}_{{\bar{Q}}_{y}}^{2}\\ & \;+\sum_{j=0}^{m-1}|\tilde{u}(j|k+1)-u_{des}-\delta_{u,k}^{*}{|}_{Q_{u}}^{2}+\sum_{j=1}^{m-1}|\Delta u^{*}(j|k){|}_{R}^{2}+|\delta_{y,k}^{*}{|}_{S_{y}}^{2}+|\delta_{u,k}^{*}{|}_{S_{u}}^{2}. \end{array}$$

Now, since $\tilde{y}(j|k+1)=y^{*}(j+1|k)$ and $\tilde{u}(j|k+1)=u^{*}(j+1|k)$ for 0≤j≤m−2, we have that


$$\begin{array}{c} {\tilde{𝒱}}_{k+1}-{𝒱}_{k}^{*}=|\tilde{y}(m-1|k+1)-y_{sp,k}^{*}-\delta_{y,k}^{*}{|}_{Q_{y}}^{2}-|y^{*}(0|k)-y_{sp,k}^{*}-\delta_{y,k}^{*}{|}_{Q_{y}}^{2}\\ +|\tilde{u}(m-1|k+1)-u_{des}-\delta_{u,k}^{*}{|}_{Q_{u}}^{2}-|u^{*}(0|k)-u_{des}-\delta_{u,k}^{*}{|}_{Q_{u}}^{2}\\ +|{\tilde{x}}_{d}(m-1|k+1){|}_{{\bar{Q}}_{y}}^{2}-|x_{d}^{*}(m-1|k){|}_{{\bar{Q}}_{y}}^{2}-|\Delta u^{*}(0|k){|}_{R}^{2}. \end{array}$$

But observe that ${\tilde{x}}_{s}(m-1|k+1)=x_{s}^{*}(m-1|k)$ and then


$$\tilde{y}(m-1|k+1)-y_{sp,k}^{*}-\delta_{y,k}^{*}=\Psi {\tilde{x}}_{d}(m-1|k+1).$$

On the other hand, we have that ${\tilde{x}}_{d}(m-1|k+1)=Fx_{d}^{*}(m-1|k)$, so that


$$|\tilde{y}(m-1|k+1)-y_{sp,k}^{*}-\delta_{y,k}^{*}{|}_{Q_{y}}^{2}+|{\tilde{x}}_{d}(m-1|k+1){|}_{{\bar{Q}}_{y}}^{2}=|x_{d}^{*}(m-1|k){|}_{{\bar{Q}}_{y}}^{2}.$$

Moreover, note that $\tilde{u}(m-1|k+1)=u^{*}(m-1|k)$ and then $\tilde{u}(m-1|k+1)-u_{des}-\delta_{u,k}^{*}=0$. Thus, we deduce that


$$\begin{array}{cc} {\tilde{𝒱}}_{k+1}-{𝒱}_{k}^{*} & =-|y^{*}(0|k)-y_{sp,k}^{*}-\delta_{y,k}^{*}{|}_{Q_{y}}^{2}-|u^{*}(0|k)-u_{des}-\delta_{u,k}^{*}{|}_{Q_{u}}^{2}-|\Delta u^{*}(0|k){|}_{R}^{2}\\ & \leq 0. \end{array}$$

Finally, since the strategy $\Delta {\tilde{u}}_{k+1}$, ${\tilde{y}}_{sp,k+1}$, ${\tilde{\delta }}_{y,k+1}$, ${\tilde{\delta }}_{u,k+1}$ at time step *k* + 1 is not necessarily the optimal one, we have that ${𝒱}_{k+1}^{*}\leq {\tilde{𝒱}}_{k+1}$ and then we conclude that the sequence $({𝒱}_{k}^{*}{)}_{k\geq 1}$ is non-increasing. □

**Lemma 5.** *Under Assumption 3, we have that*


$$\sum_{j=0}^{m-1}\Delta u^{*}(j|k)\to 0\;\text{ as }k\to \infty \text{ and }x_{d}^{*}(0|k)\to 0\;\text{ as }k\to \infty .$$

*Proof.* From the proof of the last lemma, we have that


$${𝒱}_{k+1}^{*}-{𝒱}_{k}^{*}\leq -|y^{*}(0|k)-y_{sp,k}^{*}-\delta_{y,k}^{*}{|}_{Q_{y}}^{2}-|u^{*}(0|k)-u_{des}-\delta_{u,k}^{*}{|}_{Q_{u}}^{2}-|\Delta u^{*}(0|k){|}_{R}^{2}.$$

Since the sequence $({𝒱}_{k}^{*}{)}_{k\geq 1}$ is non-increasing and non-negative, we deduce that it converges and then


$$|u^{*}(0|k)-u_{des}-\delta_{u,k}^{*}{|}_{Q_{u}}^{2}\overset{k\to \infty \;}{\to }0\text{ and }|\Delta u^{*}(0|k){|}_{R}^{2}\overset{k\to \infty \;}{\to }0.$$ (12)

Using (11), we have that


$$u^{*}(0|k)-u_{des}-\delta_{u,k}^{*}=-\sum_{j=1}^{m-1}\Delta u^{*}(j|k).$$

Thus, by (12), we obtain that


$$\sum_{j=0}^{m-1}\Delta u^{*}(j|k)\overset{k\to \infty \;}{\to }0.$$

Now, observe that


$$x_{d}^{*}(0|k+p)=F^{p}x_{d}^{*}(0|k)+\sum_{j=0}^{p-1}F^{j}D_{d}\Delta u^{*}(0|k+p-j),\text{ for }p\geq 1.$$

Under Assumption 3, for any ε>0, there exists $k_{0}\geq 1$ such that


$$\sup_{p\geq 1}|x_{d}^{*}(0|k_{0}+p)-F^{p}x_{d}^{*}(0|k_{0})|<\varepsilon /2,$$

and there exists $p_{0}\geq 1$ such that, for all $p\geq p_{0}$, $\|F^{p}x_{d}^{*}(0|k_{0})\|<\varepsilon /2$. Finally, for all $p\geq p_{0}$,


$$|x_{d}^{*}(0|k_{0}+p)|\leq |F^{p}x_{d}^{*}(0|k_{0})|+\sup_{p\geq 1}|x_{d}^{*}(0|k_{0}+p)-F^{p}x_{d}^{*}(0|k_{0})|<\varepsilon ,$$

that is, $x_{d}^{*}(0|k)\to 0$ as k→∞. □

**Lemma 6.** *Under Assumption 3, we have that*


$$\lim_{k\to \infty }{𝒱}_{k}^{*}=\lim_{k\to \infty }(\|\delta_{y,k}^{*}{\|}_{S_{y}}^{2}+\|\delta_{u,k}^{*}{\|}_{S_{u}}^{2}).$$

*Proof.* By Lemma 5, we can choose *k* large enough so that


$$\sum_{j=0}^{m-1}\Delta u^{*}(j|k)\in \Delta 𝕌.$$

For such a *k*, let $\Delta {\tilde{u}}_{k}$, ${\tilde{y}}_{sp,k}$, ${\tilde{\delta }}_{y,k}$, ${\tilde{\delta }}_{u,k}$ be such that


$$\begin{array}{cc} \Delta \tilde{u}(0|k) & =\sum_{j=0}^{m-1}\Delta u^{*}(j|k),\\ \Delta \tilde{u}(j|k) & =0,\text{ for }1\leq j<m,\\ {\tilde{y}}_{sp,k} & =y_{sp,k}^{*},\\ {\tilde{\delta }}_{y,k} & =\delta_{y,k}^{*},\\ {\tilde{\delta }}_{u,k} & =\delta_{u,k}^{*}, \end{array}$$

and observe that this is a feasible strategy for the control optimization problem at time step *k*. Indeed, we have that


$$\begin{array}{cc} x_{s}^{*}(0|k-1) & +D_{0}\sum_{j=0}^{m-1}\Delta \tilde{u}(j|k)-{\tilde{y}}_{sp,k}-{\tilde{\delta }}_{y,k}\\ \; & =x_{s}^{*}(0|k-1)+D_{0}\sum_{j=0}^{m-1}\Delta u^{*}(j|k)-y_{sp,k}^{*}-\delta_{y,k}^{*}\\ \; & =0 \end{array}$$

and


$$u^{*}(k-1)+\sum_{j=0}^{m-1}\Delta \tilde{u}(j|k)-u_{des}-{\tilde{\delta }}_{u,k}=u^{*}(k-1)+\sum_{j=0}^{m-1}\Delta u^{*}(j|k)-u_{des}-\delta_{u,k}^{*}=0,$$

so that (10) and (11) are satisfied, and we also have that


$$u^{*}(k-1)+\sum_{i=0}^{j}\Delta \tilde{u}(i|k)=u^{*}(k-1)+\sum_{i=0}^{m-1}\Delta u^{*}(i|k)\in 𝕌$$

for 0≤j<m. Moreover, note that ${\tilde{y}}_{sp,k}\in 𝕐$ and $\Delta \tilde{u}(j|k)\in \Delta 𝕌$ for 0≤j<m.

Now, the cost corresponding to the above strategy is


$$\begin{array}{c} {\tilde{𝒱}}_{k}=\sum_{j=0}^{m-1}|\tilde{y}(j|k)-y_{sp,k}^{*}-\delta_{y,k}^{*}{|}_{Q_{y}}^{2}+|{\tilde{x}}_{d}(m-1|k){|}_{{\bar{Q}}_{y}}^{2}+\sum_{j=0}^{m-1}|\tilde{u}(j|k)-u_{des}-\delta_{u,k}^{*}{|}_{Q_{u}}^{2}\\ +|\sum_{j=0}^{m-1}\Delta u^{*}(j|k){|}_{R}^{2}+|\delta_{y,k}^{*}{|}_{S_{y}}^{2}+|\delta_{u,k}^{*}{|}_{S_{u}}^{2} \end{array}$$

and, for 0≤j<m, we have that $\tilde{u}(j|k)-u_{des}-\delta_{u,k}^{*}=0$ and


$$\tilde{y}(j|k)-y_{sp,k}^{*}-\delta_{y,k}^{*}=\Psi F^{j}(Fx_{d}^{*}(0|k-1)+D_{d}\sum_{i=0}^{m-1}\Delta u^{*}(i|k)).$$

Moreover, we also have that


$${\tilde{x}}_{d}(m-1|k)=F^{m-1}(Fx_{d}^{*}(0|k-1)+D_{d}\sum_{j=0}^{m-1}\Delta u^{*}(j|k))$$

and then we obtain that


$$\begin{array}{cc} {\tilde{𝒱}}_{k} & =\sum_{j=0}^{m-1}|\Psi F^{j}(Fx_{d}^{*}(0|k-1)+D_{d}\sum_{i=0}^{m-1}\Delta u^{*}(i|k)){|}_{Q_{y}}^{2}\\ & \;+|F^{m-1}(Fx_{d}^{*}(0|k-1)+D_{d}\sum_{j=0}^{m-1}\Delta u^{*}(j|k)){|}_{{\bar{Q}}_{y}}^{2}\\ & \;+|\sum_{j=0}^{m-1}\Delta u^{*}(j|k){|}_{R}^{2}+|\delta_{y,k}^{*}{|}_{S_{y}}^{2}+|\delta_{u,k}^{*}{|}_{S_{u}}^{2}\\ & =|Fx_{d}^{*}(0|k-1)+D_{d}\sum_{j=0}^{m-1}\Delta u^{*}(j|k){|}_{E}^{2}+|\sum_{j=0}^{m-1}\Delta u^{*}(j|k){|}_{R}^{2}+|\delta_{y,k}^{*}{|}_{S_{y}}^{2}+|\delta_{u,k}^{*}{|}_{S_{u}}^{2} \end{array}$$

where


$$E=\sum_{j=0}^{m-1}(\Psi F^{j}{)}^{T}Q_{y}(\Psi F^{j})+(F^{m-1}{)}^{T}{\bar{Q}}_{y}F^{m-1}=\sum_{j=0}^{\infty }(\Psi F^{j}{)}^{T}Q_{y}(\Psi F^{j})=\Psi^{T}Q_{y}\Psi +{\bar{Q}}_{y}.$$

Hence, by Lemma 5, we deduce that


$${\tilde{𝒱}}_{k}-(\|\delta_{y,k}^{*}{\|}_{S_{y}}^{2}+\|\delta_{u,k}^{*}{\|}_{S_{u}}^{2})\overset{k\to \infty \;}{\to }0.$$

On the other hand, note that ${𝒱}_{k}^{*}\leq {\tilde{𝒱}}_{k}$ and ${𝒱}_{k}^{*}-(\|\delta_{y,k}^{*}{\|}_{S_{y}}^{2}+\|\delta_{u,k}^{*}{\|}_{S_{u}}^{2})\geq 0$, therefore


$${𝒱}_{k}^{*}-(\|\delta_{y,k}^{*}{\|}_{S_{y}}^{2}+\|\delta_{u,k}^{*}{\|}_{S_{u}}^{2})\overset{k\to \infty \;}{\to }0.$$

Finally, since the sequence $({𝒱}_{k}^{*}{)}_{k\geq 1}$ converges, we conclude that


$$\lim_{k\to \infty }{𝒱}_{k}^{*}=\lim_{k\to \infty }(\|\delta_{y,k}^{*}{\|}_{S_{y}}^{2}+\|\delta_{u,k}^{*}{\|}_{S_{u}}^{2}).$$

As a byproduct of the last lemma, we have the following □

**Corollary 1.** *Under the same assumption of Lemma 6, we have that*


$$\sum_{j=0}^{m-1}|\Delta u^{*}(j|k){|}_{R}^{2}\to 0\;as\;k\to \infty .$$

### Proof of Theorem 3

Assume that $S_{u}>H+I_{n_{u}}$, where $I_{n_{u}}$ is the identity matrix of dimension *n*_*u*_ and


$$H=(m-1)D_{0}^{T}Q_{y}D_{0}+(\Psi D_{d}{)}^{T}Q_{y}(\Psi D_{d})+D_{d}^{T}{\bar{Q}}_{y}D_{d}+(m-1)Q_{u}+R,$$

and suppose that $\lim_{k\to \infty }{𝒱}_{k}^{*}>0$.

By Corollary 1, we can choose a large enough *k* such that


$$\Delta u^{*}(m-1|k)\in \text{int}\;\Delta 𝕌.$$ (13)

For such a *k*, let α=α(k)∈(0,1) be such that


$$\Delta u^{*}(m-1|k)-(1-\alpha )\delta_{u,k}^{*}\in \Delta 𝕌$$

and consider the following strategy for the control optimization problem at time step *k*:


$$\begin{array}{cc} \Delta \tilde{u}(j|k) & =\Delta u^{*}(j|k),\text{ for }0\leq j<m-1,\\ \Delta \tilde{u}(m-1|k) & =\Delta u^{*}(m-1|k)-(1-\alpha )\delta_{u,k}^{*},\\ {\tilde{y}}_{sp,k} & =\alpha y_{sp,k}^{*}+(1-\alpha )D_{0}u_{des},\\ {\tilde{\delta }}_{y,k} & =\alpha \delta_{y,k}^{*},\\ {\tilde{\delta }}_{u,k} & =\alpha \delta_{u,k}^{*}. \end{array}$$

It is straightforward to check that this strategy satisfies (11) and, from the fact that the solution of the control optimization problem at time step *k* satisfies (10) (11), we deduce that


$$D_{0}(u_{des}+\delta_{u,k}^{*})=y_{sp,k}^{*}+\delta_{y,k}^{*}$$

which can be used to check that the above strategy also satisfies (10). Moreover, we have that $\Delta \tilde{u}(j|k)\in \Delta 𝕌$, for 0≤j<m, and


$$u^{*}(k-1)+\sum_{i=0}^{j}\Delta \tilde{u}(i|k)\in 𝕌,\text{ for }0\leq j<m-1.$$

Using the fact that $\delta_{u,k}^{*}=u^{*}(m-1|k)-u_{des}$, which follows from (11), we also have that


$$u^{*}(k-1)+\sum_{i=0}^{m-1}\Delta \tilde{u}(i|k)=\alpha u^{*}(m-1|k)+(1-\alpha )u_{des}\in 𝕌,$$

since both $u^{*}(m-1|k)\in 𝕌$ and $u_{des}\in 𝕌$. Also, note that ${\tilde{y}}_{sp,k}\in 𝕐$ since both $y_{sp,k}^{*}\in 𝕐$ and $D_{0}u_{des}\in 𝕐$. Thus, the above strategy is indeed feasible at time step *k*, and the corresponding cost is


$$\begin{array}{c} {\tilde{𝒱}}_{k}=\sum_{j=0}^{m-1}|y^{*}(j|k)-y_{sp,k}^{*}-\delta_{y,k}^{*}{|}_{Q_{y}}^{2}+(m-1)(1-\alpha {)}^{2}|D_{0}\delta_{u,k}^{*}{|}_{Q_{y}}^{2}+(1-\alpha {)}^{2}|\Psi D_{d}\delta_{u,k}^{*}{|}_{Q_{y}}^{2}\\ +2(1-\alpha )(\sum_{j=0}^{m-2}‹y^{*}(j|k)-y_{sp,k}^{*}-\delta_{y,k}^{*},D_{0}\delta_{u,k}^{*}{›}_{Q_{y}}-‹\Psi x_{d}^{*}(m-1|k),\Psi D_{d}\delta_{u,k}^{*}{›}_{Q_{y}})\\ +|x_{d}^{*}(m-1|k){|}_{{\bar{Q}}_{y}}^{2}+(1-\alpha {)}^{2}|D_{d}\delta_{u,k}^{*}{|}_{{\bar{Q}}_{y}}^{2}-2(1-\alpha )‹x_{d}^{*}(m-1|k),D_{d}\delta_{u,k}^{*}{›}_{{\bar{Q}}_{y}}\\ +\sum_{j=0}^{m-1}|u^{*}(j|k)-u_{des}-\delta_{u,k}^{*}{|}_{Q_{u}}^{2}+(m-1)(1-\alpha {)}^{2}|\delta_{u,k}^{*}{|}_{Q_{u}}^{2}\\ +2(1-\alpha )\sum_{j=0}^{m-2}‹u^{*}(j|k)-u_{des}-\delta_{u,k}^{*},\delta_{u,k}^{*}{›}_{Q_{u}}\\ +\sum_{j=0}^{m-1}|\Delta u^{*}(j|k){|}_{R}^{2}+(1-\alpha {)}^{2}|\delta_{u,k}^{*}{|}_{R}^{2}-2(1-\alpha )‹\Delta u^{*}(m-1|k),\delta_{u,k}^{*}{›}_{R}\\ +\alpha^{2}|\delta_{y,k}^{*}{|}_{S_{y}}^{2}+\alpha^{2}|\delta_{u,k}^{*}{|}_{S_{u}}^{2}. \end{array}$$

Hence, we have that


$$\begin{array}{c} \frac{{\tilde{𝒱}}_{k}-{𝒱}_{k}^{*}}{1-\alpha }=2(\sum_{j=0}^{m-2}‹y^{*}(j|k)-y_{sp,k}^{*}-\delta_{y,k}^{*},D_{0}\delta_{u,k}^{*}{›}_{Q_{y}}-‹\Psi x_{d}^{*}(m-1|k),\Psi D_{d}\delta_{u,k}^{*}{›}_{Q_{y}}\\ -‹x_{d}^{*}(m-1|k),D_{d}\delta_{u,k}^{*}{›}_{{\bar{Q}}_{y}}+\sum_{j=0}^{m-2}‹u^{*}(j|k)-u_{des}-\delta_{u,k}^{*},\delta_{u,k}^{*}{›}_{Q_{u}}\\ -‹\Delta u^{*}(m-1|k),\delta_{u,k}^{*}{›}_{R})\\ -(1+\alpha )|\delta_{y,k}^{*}{|}_{S_{y}}^{2}+(1-\alpha )|\delta_{u,k}^{*}{|}_{H}^{2}-(1+\alpha )|\delta_{u,k}^{*}{|}_{S_{u}}^{2}. \end{array}$$

Now, if ${lim sup}_{k\to \infty }\|\delta_{u,k}^{*}{\|}_{S_{u}}^{2}>0$ then by equivalence of the norms, we have that

${lim sup}_{k\to \infty }\|\delta_{u,k}^{*}{\|}^{2}>0$ (where ‖·‖ is the euclidean norm) and therefore there exists *c* > 0 such that $\|\delta_{u,k}^{*}{\|}^{2}>c$ infinitely often. Since


$$x_{d}^{*}(m-1|k)=F^{m}x_{d}^{*}(0|k-1)+\sum_{i=0}^{m-1}F^{m-1-i}D_{d}\Delta u^{*}(i|k)$$ (14)

and, for 0≤j≤m−2,


$$y^{*}(j|k)-y_{sp,k}^{*}-\delta_{y,k}^{*}=\Psi [F^{j+1}x_{d}^{*}(0|k-1)+\sum_{i=0}^{j}F^{j-i}D_{d}\Delta u^{*}(i|k)]-D_{0}\sum_{i=j+1}^{m-1}\Delta u^{*}(i|k)$$ (15)

and also


$$u^{*}(j|k)-u_{des}-\delta_{u,k}^{*}=-\sum_{i=j+1}^{m-1}\Delta u^{*}(i|k),$$ (16)

by Lemma 5 and Corollary 1, for 0<ε<c, there exists *k*_0_ (large enough) satisfying (13) such that


$$\frac{{\tilde{𝒱}}_{k_{0}}-{𝒱}_{k_{0}}^{*}}{1-\alpha (k_{0})}<\varepsilon +\|\delta_{u,k_{0}}^{*}{\|}_{H}^{2}-\|\delta_{u,k_{0}}^{*}{\|}_{S_{u}}^{2}<\varepsilon -\|\delta_{u,k_{0}}^{*}{\|}^{2}<\varepsilon -c,$$

since $S_{u}>H+I_{n_{u}}$. Finally, as ε−c<0, we conclude that ${\tilde{𝒱}}_{k_{0}}<{𝒱}_{k_{0}}^{*}$, which is a contradiction.

On the other hand, if ${lim sup}_{k\to \infty }\|\delta_{u,k}^{*}{\|}_{S_{u}}^{2}=0$ then, by Lemma 6, we have that


$$\lim_{k\to \infty }\|\delta_{y,k}^{*}{\|}_{S_{y}}^{2}=\lim_{k\to \infty }{𝒱}_{k}^{*}>0,$$

and using (14), (15), (16), Lemma 5 and Corollary 1 we deduce that, for $0<\varepsilon^{\prime }<\lim_{k\to \infty }{𝒱}_{k}^{*}$, there exists *k*_1_ (large enough) satisfying (13) such that


$$\frac{{\tilde{𝒱}}_{k_{1}}-{𝒱}_{k_{1}}^{*}}{1-\alpha (k_{1})}<\frac{\varepsilon^{\prime }}{2}-\|\delta_{y,k_{1}}^{*}{\|}_{S_{y}}^{2}<\frac{\varepsilon^{\prime }}{2}-(\lim_{k\to \infty }{𝒱}_{k}^{*}-\frac{\varepsilon^{\prime }}{2})=\varepsilon^{\prime }-\lim_{k\to \infty }{𝒱}_{k}^{*}<0$$

and then ${\tilde{𝒱}}_{k_{1}}<{𝒱}_{k_{1}}^{*}$, which is a contradiction once again. □

### Proof of Theorem 4

Consider the following strategy for the control optimization problem at time step *k* = 1,


$$\begin{array}{cc} \Delta \tilde{u}(j|1) & =0,\text{ for }0\leq j<m,\\ {\tilde{y}}_{sp,1} & =D_{0}u_{des},\\ {\tilde{\delta }}_{y,1} & =-D_{0}u_{des},\\ {\tilde{\delta }}_{u,1} & =-u_{des}. \end{array}$$

Since the system is assumed to be initially in the steady state u(0)=0, $x_{s}(0)=0$, $x_{d}(0)=0$, the above strategy is indeed feasible and it has an associated cost given by


$${\tilde{𝒱}}_{1}=\|D_{0}u_{des}{\|}_{S_{y}}^{2}+\|u_{des}{\|}_{S_{u}}^{2}.$$

Moreover, since this strategy is not necessarily the optimal one, and using Lemma 4, we have that ${𝒱}_{k}^{*}\leq {𝒱}_{1}^{*}\leq {\tilde{𝒱}}_{1}$ for all k≥1.

Now, for any k≥1, we have that


$$\|y^{*}(0|k)-y_{sp,k}^{*}-\delta_{y,k}^{*}{\|}_{Q_{y}}^{2}+\|\delta_{y,k}^{*}{\|}_{S_{y}}^{2}+\|u^{*}(0|k)-u_{des}-\delta_{u,k}^{*}{\|}_{Q_{u}}^{2}+\|\delta_{u,k}^{*}{\|}_{S_{u}}^{2}\leq {𝒱}_{k}^{*}.$$

On the other hand,


$$\begin{array}{cc} \|y^{*}(0|k)-y_{sp,k}^{*}{\|}_{Q_{y}}^{2} & \leq 2(\|y^{*}(0|k)-y_{sp,k}^{*}-\delta_{y,k}^{*}{\|}_{Q_{y}}^{2}+\|\delta_{y,k}^{*}{\|}_{Q_{y}}^{2})\\ & \leq C_{1}(\|y^{*}(0|k)-y_{sp,k}^{*}-\delta_{y,k}^{*}{\|}_{Q_{y}}^{2}+\|\delta_{y,k}^{*}{\|}_{S_{y}}^{2}) \end{array}$$

for some positive constant *C*_1_ and


$$\begin{array}{cc} \|u^{*}(0|k)-u_{des}{\|}_{Q_{u}}^{2} & \leq 2(\|u^{*}(0|k)-u_{des}-\delta_{u,k}^{*}{\|}_{Q_{u}}^{2}+\|\delta_{u,k}^{*}{\|}_{Q_{u}}^{2})\\ & \leq C_{2}(\|u^{*}(0|k)-u_{des}-\delta_{u,k}^{*}{\|}_{Q_{u}}^{2}+\|\delta_{u,k}^{*}{\|}_{S_{u}}^{2}) \end{array}$$

for some positive constant *C*_2_. Thus, we obtain


$$\left|[\begin{array}{c} y^{*}(0|k)-y_{sp,k}^{*}\\ u^{*}(0|k)-u_{des} \end{array}]|=(\|y^{*}(0|k)-y_{sp,k}^{*}{\|}^{2}+\|u^{*}(0|k)-u_{des}{\|}^{2}{)}^{1/2}\leq C_{3}\|u_{des}\right\|$$

for some positive constant *C*_3_, for all k≥1, which finally implies the result. □

## Conclusion

The main contribution of this paper is to provide a rigorous mathematical analysis for the asymptotic stability, i.e., convergence and stability, of two important approaches in Model Predictive Control (MPC) based on the Output Prediction-Oriented Model (OPOM): the EIHMPC developed by [4] and the EIHMPC with zone control [5]. The key contributions include:

1. Rigorous proof for the EIHMPC: The first objective was to provide a rigorous proof of asymptotic stability for this controller. This analysis significantly extends prior works and develops mathematical proofs using geometric and algebraic tools that are valid for any input horizon *m* and general gain matrix *D*_0_, including singular matrices. The results confirm convergence (Theorem 1), showing that the optimal cost function converges to zero as time tends to infinity, provided the matrix *S* is correctly chosen. The results also confirm nominal stability (Theorem 2), demonstrating that if the output reference *r* is sufficiently close to the starting point of the process, the optimal output deviation remains small for all time steps. We already emphasized that the EIHMPC is not a particular case of the EIHMPC with zone control, justifying separate mathematical proofs for both controllers.
2. Rigorous proof for the EIHMPC with zone control: In the same spirit, this paper developed a rigorous proof of asymptotic stability for the EIHMPC with zone control, considering any input horizon *m* and general gain matrix *D*_0_. Our analysis established convergence (Theorem 3), showing that the optimal cost function converges to zero if the tuning parameter matrix *S*_*u*_ is well chosen. It also established nominal stability (Theorem 4), concluding that if the external input target *u*_*des*_ is close enough from the starting point of the process, the deviation in output and input variables remains small for all time steps.
3. Practical implementation enhancements: As a byproduct of the mathematical analysis, we provided implementable expressions for tunning parameters *S* of the EIHMPC (see the proof of Proposition 1 and the Appendix) and *S*_*u*_ of the EIHMPC with zone control (see Theorem 3).
4. Mathematical background: The proofs presented in this work are also intended to provide a mathematical foundation to study asymptotic stability in related nominal MPC frameworks (e.g., RTO-MPC integration, multi-model adaptive RTO-MPC integration). Another natural direction for future work would be, for example, to extend the convergence and stability results to corresponding robust controllers.

### Appendix

In this appendix, we first obtain an explicit expression for the constant *C*_3_ that appears in the proof of Proposition 1. For a positive semidefinite matrix *A*, let us define


$$\Gamma_{A}^{2}:=\sup_{\|x\|=1}x^{T}Ax,$$

where ‖·‖ is the euclidean norm. It is well known that $\Gamma_{A}^{2}$ is the spectral radius of *A*. Starting back from the first inequality of (9), we have that the term


$$\begin{array}{cc} \sum_{j=0}^{m-1}\|\Psi F^{j+1} & x_{d}^{*}(0|k-1){\|}_{Q}\|\Psi F^{j}D_{d}\Delta \tilde{u}(0|k){\|}_{Q}+\|F^{m}x_{d}^{*}(0|k-1){\|}_{\bar{Q}}\|F^{m-1}D_{d}\Delta \tilde{u}(0|k){\|}_{\bar{Q}}\\ & \leq (\sum_{j=0}^{m-1}\|\Psi F^{j+1}x_{d}^{*}(0|k-1){\|}_{Q}+\|F^{m}x_{d}^{*}(0|k-1){\|}_{\bar{Q}})\\ & \;\;\;\;\;\;\;\;\;\;\;\;\;\;\;\;\;\;\times (\sum_{j=0}^{m-1}\|\Psi F^{j}D_{d}\Delta \tilde{u}(0|k){\|}_{Q}+\|F^{m-1}D_{d}\Delta \tilde{u}(0|k){\|}_{\bar{Q}})\\ & =\|x_{d}^{*}(0|k-1){\|}_{\bar{Q}}\|\Delta \tilde{u}(0|k){\|}_{Z-R}\\ & \leq \Gamma_{\bar{Q}}\Gamma_{Z-R}\|x_{d}^{*}(0|k-1)\|\|\Delta \tilde{u}(0|k)\|, \end{array}$$

and therefore we obtain that


$$C_{3}=2\varphi^{-2}[\Gamma_{Z}^{2}\vee (2\Gamma_{\bar{Q}}\Gamma_{Z-R})],$$ (17)

where φ and *Z* are defined in the proof of Proposition 1.

Next, we give a recipe to compute the matrix *S* from Theorem 1. This is done in three steps:

1. From matrix *D*_0_, compute the matrix $\hat{S}=K_{1}^{T}(L^{-1}{)}^{T}L^{-1}K_{1}+K_{2}^{T}K_{2}$ (see the paragraph just above Proposition 1 for the definitions of *K*_1_, *K*_2_ and *L*). Observe that in the case *D*_0_ regular, the expression of $\hat{S}$ boils down to $\hat{S}=(D_{0}^{-1}{)}^{T}D_{0}^{-1}$.
2. Next, compute (or obtain an upper bound for) *C*_3_ using expression (17).
3. Finally, choose $\beta >6C_{3}$ and set $S=\beta \hat{S}$.

Now, in order to illustrate the above recipe, we present the following toy example. Consider a system with *n*_*u*_ = 3, $n_{y}=n_{d}=2$, $𝕌=[0,1{]}^{3}$, *u*_*r*_ = (1/2,1/2,1/2) and


$$\begin{array}{c} F=[\begin{array}{cc} 1/2 & 0\\ 0 & 1/3 \end{array}],\;D_{0}=[\begin{array}{ccc} -3 & 1 & 1\\ -4 & 1 & 2 \end{array}],\;D_{d}=[\begin{array}{ccc} 3 & 0 & -1\\ 2 & -1 & 2 \end{array}],\;\Psi =[\begin{array}{cc} 1/2 & 1/2\\ 1/3 & 2/3 \end{array}]. \end{array}$$

Also, consider the input horizon *m* = 2 and weighting matrices *Q* = *I*_2_ and *R* = *I*_3_, i.e., the identity matrices of dimensions 2 and 3, respectively.

From *D*_0_, 𝕌 and *u*_*r*_, we deduce the following:


$$\begin{array}{cc} \ker D_{0} & =\text{span}\{(1,2,1)\},\\ (\ker D_{0}{)}^{⟂} & =\text{span}\{(1,-1,1),(1,0,-1)\},\\ \text{Im}D_{0} & =\text{span}\{(1,0),(0,1)\}={\mathbb{R}}^{2},\\ (\text{Im}D_{0}{)}^{⟂} & =\{0\},\\ \varphi =\cos\theta_{\left(0,0,0\right)} & =\frac{‹(\frac{1}{6},-\frac{1}{6},\frac{1}{6}),(\frac{1}{4},0,\frac{1}{4})›}{\left|(\frac{1}{6},-\frac{1}{6},\frac{1}{6})||(\frac{1}{4},0,\frac{1}{4})\right|}=\sqrt{\frac{2}{3}}. \end{array}$$

1. Computation of *Ŝ*:

We first obtain the matrix *M* of $D_{0}^{⟂}$ in the orthonormal bases of $(\ker D_{0}{)}^{⟂}$ and Im*D*_0_ (see the paragraph just above Proposition 1):


$$\begin{array}{c} M=[\begin{array}{cc} -\sqrt{3} & -2\sqrt{2}\\ -\sqrt{3} & -3\sqrt{2} \end{array}]. \end{array}$$

The *LQ*-factorization *M* = *LO*, with lower triangular *L* and orthogonal *O*, is given by


$$\begin{array}{c} L=[\begin{array}{cc} \sqrt{11} & 0\\ \frac{15}{\sqrt{11}} & \sqrt{\frac{6}{11}} \end{array}]\text{ and }O=[\begin{array}{cc} -\sqrt{\frac{3}{11}} & -2\sqrt{\frac{2}{11}}\\ 2\sqrt{\frac{2}{11}} & -\sqrt{\frac{3}{11}} \end{array}]. \end{array}$$

We also deduce that the matrix *K*_1_ is simply the two-dimensional identity, while *K*_2_ is scalar null. Thus, we have that


$$\begin{array}{c} \hat{S}=(L^{-1}{)}^{T}L^{-1}=[\begin{array}{cc} \frac{7}{2} & -\frac{5}{2}\\ -\frac{5}{2} & \frac{11}{6} \end{array}]. \end{array}$$

2. Upper bound for *C*_3_:

Now, we obtain the matrix


$$\begin{array}{c} \bar{Q}=[\begin{array}{cc} \frac{13}{108} & \frac{17}{180}1.50mm\\ \frac{17}{180} & \frac{25}{288} \end{array}], \end{array}$$

which is the solution to the Lyapunov equation $\bar{Q}-F^{T}\bar{Q}F=F^{T}\Psi^{T}Q\Psi F$. Next, we compute the matrix *Z* from (8),


$$\begin{array}{c} Z=[\begin{array}{ccc} \frac{1349}{72} & -\frac{379}{72} & \frac{1609}{360}1.50mm\\ -\frac{3007}{720} & \frac{563}{288} & -\frac{277}{240}\;1.50mm\\ \frac{209}{40} & -\frac{899}{720} & \frac{559}{216} \end{array}]. \end{array}$$

The spectral radii of *Z*, $\bar{Q}$ and Z−R are respectively $\Gamma_{Z}^{2}\approx 20.01$, $\Gamma_{\bar{Q}}^{2}\approx 0.19$ and $\Gamma_{Z-R}^{2}\approx 19.01$. Thus, we obtain that


$$C_{3}<2\frac{3}{2}[21\vee (2\sqrt{0.2}\sqrt{20})]=63.$$

3. Deduction of *S*:

Finally, we choose $\beta =6\times 63=378>6C_{3}$, which gives


$$\begin{array}{c} S=378[\begin{array}{cc} \frac{7}{2} & -\frac{5}{2}\\ -\frac{5}{2} & \frac{11}{6} \end{array}]. \end{array}$$

### List of symbols

*D*_0_, *D*_*d*_, *F*, Ψ: matrices of the OPOM (see (1) and (2));

*x*_*s*_: static part of the state of the OPOM;

*x*_*d*_: dynamic part of the state of the OPOM;

*u*: input variable of the OPOM;

Δu: input increment variable of the OPOM;

*y*: output variable of the OPOM;

*m*: input horizon for both controllers;

*V*: cost function of the EIHMPC;

*Q*, *S*: weighting matrices of the EIHMPC;

δ: slack variable of the EIHMPC;

*r*: set-point of the EIHMPC;

𝒱: cost function of the EIHMPC with zone control;

*Q*_*u*_, *Q*_*y*_, *S*_*u*_, *S*_*y*_: weighting matrices of the EIHMPC with zone control;

$\delta_{u}$, $\delta_{y}$: slack variables of the EIHMPC with zone control;

*u*_*des*_: input target of the EIHMPC with zone control;

*y*_*sp*_: set-point of the EIHMPC with zone control;

*R*: input increment weighting matrix for both controllers;

𝕌: input domain for both controllers;

Δ𝕌: input increment domain for both controllers;

𝕐: set-point domain for the EIHMPC with zone control.
